# Particle Depositions and Related Hemodynamic Parameters in the Multiple Stenosed Right Coronary Artery

**DOI:** 10.4021/jocmr843w

**Published:** 2012-05-15

**Authors:** Sandor I. Bernad, Elena S. Bernad, Marius Craina, Izabella Sargan, Alin Totoran, Cosmin Brisan

**Affiliations:** a Centre for Fundamental and Advanced Research, Romanian Academy - Timisoara Branch, Timisoara, Romania; b “Bega” Education and Research Hospital, University of Medicine and Pharmacy “Victor Babes” Timisoara, Romania; c Department of Anatomy, University of Medicine and Pharmacy “Victor Babes” Timisoara, Romania; d Department of Biomedical Engineering, Politehnica University of Timisoara, Romania

**Keywords:** Flow separation, Pressure drop, Wall shear stress, Numerical simulation

## Abstract

**Background:**

Blood flow analysis of the human right coronary artery (RCA) has been carried out to investigate the effects of serial stenosis on coronary hemodynamics. A 3-D model of a serial stenosed RCA was reconstructed based on multislice computerized tomography images.

**Methods:**

A velocity waveform in the proximal RCA and a pressure waveform in the distal RCA of a patient with a severe stenosis were acquired with a catheter delivered wire probe and applied as boundary conditions. The numerical analysis examines closely the effect of a multiple serial stenosis on the hemodynamic characteristics such as flow separation, wall shear stress (WSS) and particle depositions.

**Results and Conclusions:**

Energy loss associated with such flow expansion after each constriction will be large and consequently the pressure drop will be higher. Overall pressure drop increased from 1700 Pa (12.75 mmHg) at the end diastole to 11000 Pa (82.5 mmHg) at the peak systole. At the peak systole the WSS values reached 110 Pa in the stenosis with 28% diameter reduction and 210 Pa in the stenosis with 54% diameter reduction, which is high enough to damage the endothelial cells. However at the end of one cardiac cycle a percent of 1.4% (15 from 1063 particles release at the inlet section) remain inside the stenosed RCA.

## Introduction

The challenge of experimental and numerical investigations on the blood flow in stenotic arteries are the non-Newtonian rheology of blood, the compliant characteristic of arterial wall, the wall composition in layers, the pulsatile inlet flow determination, the mass transfer process, the geometry of the stenosis, and the transition to unsteady turbulent flow. The unsteady flow in a stenotic artery is characterized by high pressure and wall shear stress (WSS) at the throat and a recirculation zone distal to the stenosis [1, 2].

Significant atherosclerotic stenosis produces epicardial conduit resistance. In response to the loss of perfusion pressure and flow to the distal (poststenotic) vascular bed, the small resistance vessels dilate to maintain satisfactory basal flow appropriate for myocardial oxygen demand [3]. Viscous friction, flow separation forces, and flow turbulence at the site of the stenosis produce energy loss at the stenosis. Energy is extracted reducing pressure distal to the stenosis, producing a pressure gradient between proximal and distal artery regions [4, 5].

Multiple stenoses in diseased vascular bed may occur because of the formation of the primary stenosis that can result in downstream circulation flow. As a result of the secondary stenosis, a circulation zone will form at its downstream, thus resulting in a third stenosis, etc. The effects of these stenoses result in a series of sequence constrictions. Talukder et al [6], Van Dreumel and Kuiken [7] have carried out experimental studies related to flow dynamics in doubly constricted vessels. They suggested that the flow energy loss due to the presence of the stenoses, which is directly related to the pressure drops across them, increases with the number of stenoses and is not strongly dependent on the spacing between them. Lee [8] and Damodaran [9] have made 2D steady flow computational analysis of flow in multiply constricted vessels. Kilpatrick et al [10] presented an approximate assessment of the combined effect by summing the value of the resistance for each stenoses, but not by the degree of the stenoses. Gould and Lipscomb [11], Sabbah and Stein [12] concluded that multiple stenoses produce more resistance to flow than a single stenosis of similar length. Johnston and Kilpatrick [13] simulated the arterial blood flow in paired smooth stenoses and triple smooth stenoses respectively. They concluded that the more severe stenosis dominates the pair and the recirculation between stenoses is stronger with a severe proximal stenosis than a severe distal stenosis. Bertolotti et al [14] studied the influences of multiple stenoses numerically as well as experimentally to diagnosis peripheral arterial diseases and evaluated the peak systolic velocity ratio and pressure drop to detect and grade multiple stenoses in lower limb mimicking arteries.

Within the scope of this paper, we will cover the fundamental aspects of the multiple stenosis of the right coronary artery (RCA), non-invasive assessment of coronary flow and pressure, and their utilization in clinical practice to facilitate decision making within the cardiac catheterization laboratory.

## Methods

Hemodynamic flow assessment in coronary arteries is usually performed with intravascular Doppler ultrasound by measuring local velocities [15]. An alternative means for invasive flow measurements is presented by the calculation of models in which blood flow can be virtually simulated, a method that is called computational fluid dynamics (CFD). In fact, several in vitro studies [16-20] have shown that CFD allows reliable physiologic blood flow simulation and measurements of WSS, wall pressure, and mass flow.

### RCA geometry

**Figure 1:**
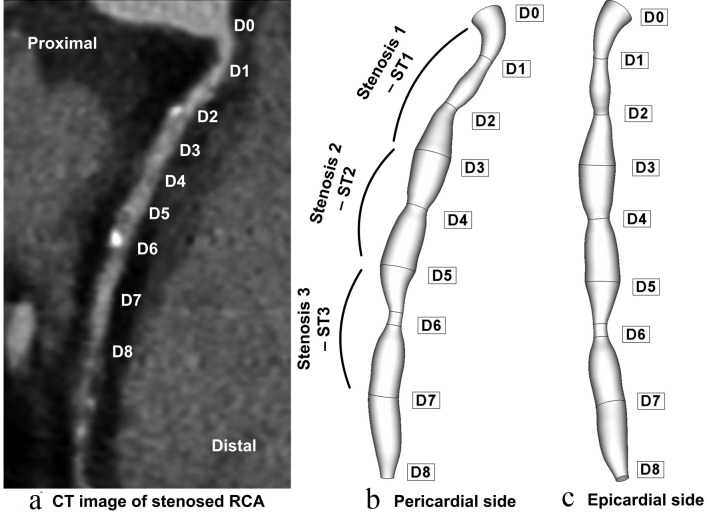
Patient-specific right coronary artery (RCA); (a) axial tomographic image which indicates a multiple coronary stenosis at the right coronary artery; geometry reconstruction (b) pericardial side, (c) epicardial side.

For the case presented in this paper, spiral CT (computed tomography) was performed for 4 days following the CA (coronary angiography) (44 year old, patient with typical angina symptoms is investigated). A Somatom Sensation 64 Scanner (Siemens Medical Systems, Erlangen, Germany) was used in non-enhanced spiral scan technique with a slice thickness of 2 mm, a table feed of 3 mm/s, and an increment of 2 mm. Data corresponding to the investigated patient is presented in Table 1. The CA and the spiral CT investigation detected a multiple severe right coronary artery (RCA) stenosis (Fig. 1). According to the NASCET (North American Symptomatic Carotid Endarterectomy Trial) [21] and ECST (European Carotid Surgery Trial) [22] method of stenosis classification, the stenoses severity is illustrated in Table 2. The percentage diameter reduction for a circular stenosis is 1-d/D, where d is the diameter of the lumen and D the diameter of the unoccluded artery.

**Table 1:** Preoperative Patient Characteristics

Variables	Value
Mean age	44
Gender (M/F)	Male
History of MI	NO
Previous PTCA	NO
Renal insufficiency	NO
Cardiovascular risk factor	
Hypertension	Yes
Diabetes	NO
Smoke	Yes
Obesity	Moderate
Angiographic data	
RCA stenosis	multiple/severe

MI = myocardial infarction, PTCA = percutaneous transluminal coronary angioplasty, RCA = right coronary artery

**Table 2:** RCA Stenosis Severity

Stenosis	Reference diameter (mm)	Stenosis minimal diameter (mm)	Stenosis length (mm)	Diameter reduction percentage (%)	Area reduction percentage (%)
ST1	3.8	1.76	12	54	77
ST2	3.6	2.6	7	28	53
ST3	3.6	1.68	7	53	80

Figure 1 shows the reconstructed lumen geometry of the RCA viewed from the epicardial, and pericardial sides. The lumen inlet diameter (at the ostium) of the RCA was 3.8 mm. The RCA is modeled to be 52 mm in length with variable diameters, depending on the stenosis severity. Data corresponding to the reconstructed RCA is presented in Table 2. Good agreement has been demonstrated between coronary artery diameter measurements in the present case (3.6 mm) and the measurements done by trans thoracic echocardiography (TTE) and quantitative coronary angiography (QCA) (3.6 ± 0.42 mm) presented by Kiviniemi et al [23].

An effective meshing procedure in this study is one that can mesh the CT scan image surface geometry without compromising the geometric features. To achieve this task the commercial grid generation software, GAMBIT 2.4.6 (ANSYS FLUENT, ANSYS, Inc.) was used [24].

The elements employed to mesh the 3D computational domain of the coronary arterial segment, consisted primarily of regular structured hexahedral elements as well as wedge elements wherever necessary. In order to carry out the mesh sensitivity analysis, numerical simulations were carried out by varying the number of mesh elements in the computational domain. The accuracy of the simulation results was then improved by employing a finer mesh that contained 1,598,752 elements.

### Computational fluid dynamics

The blood is assumed to be incompressible, with a Newtonian behavior having dynamic viscosity (μ) of 0.00408 Pa and a density (ρ) of 1050 kg/m^3^. Johnston et al. [19] compared the effects of different blood viscosity models on the WSS distributions in the RCA during the cardiac cycle. Their study showed that the use of a Newtonian blood model is a reasonably good approximation when studying the WSS distribution for transient blood flow in arteries.

The low Reynolds number k-ε model was used in this study to solve the time dependent 3D Navier-Stokes equations for an incompressible viscous fluid were applied in the numerical analysis. The blood flow is simulated with the commercial CFD FLUENT 6.3 package [24], parallelized across eight 3.2 GHz Intel processors of a TYANPSC T-650 Rx (Tyan Computer Corporation, Taiwan, parallel computing machine with sixteen 3.2 GHz processors) is used in order to perform the numerical analysis.

**Figure 2:**
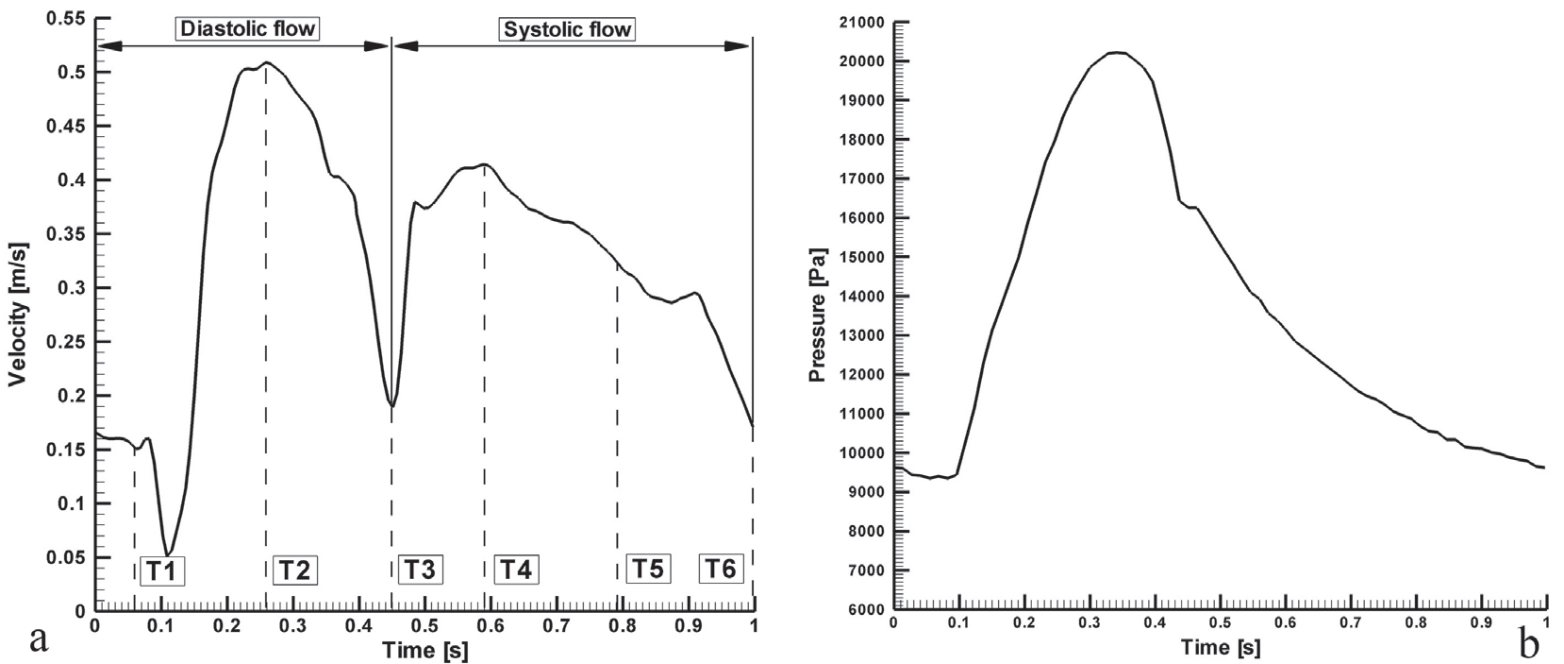
Pulsatile inlet flow, which is the physiologically realistic case, based on flow and pressure waveforms acquired with an intravascular ultrasound Doppler probe in the RCA [26]. (a) velocity input waveform; (b) pressure output waveform.

The boundary conditions required to solve the governing equations are as follows. The walls were taken as solid and stiff, and a zero-velocity boundary condition was assumed for the walls, corresponding to a no-slip condition. The inlet is identical to the real coronary ostium and every point at the inlet has identical flow parameters, including direction and velocity. The flow velocity profile at the different inlets was based on standard data reflecting the physiologically pulsatile, biphasic blood flow from the ascending aorta into the coronary arteries (Fig. 2) [25].

The calculated flow variables were flow velocities, wall shear stress (WSS), and wall pressure. The TECPLOT (Tecplot, Inc, Bellevue, WA) software (Version 10.0) was used for the visualization of flow patterns, the quantification of WSS and wall pressure, and for the measurement of mass flow at selected sites.

### Code validation

**Figure 3:**
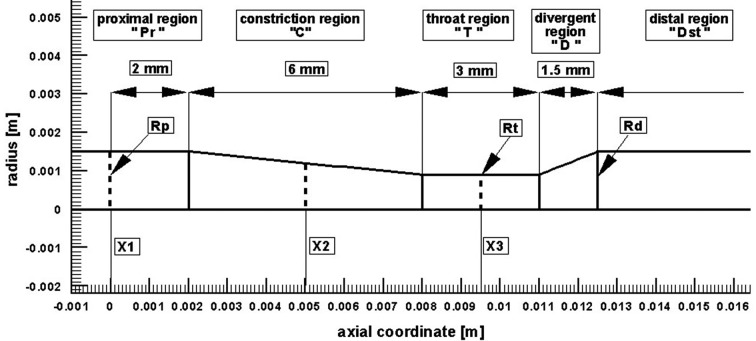
Computational geometry used for code validation [26].

Before presenting the main findings, we first validate our numerical tool against the problem investigated experimentally by Banerjee et al [26]. The case considered is the unsteady pulsatile flow, incompressible, Navier-Stokes flow through an axisymmetric moderate restenosis after percutaneous transluminal balloon coronary angioplasty (PTCA). The geometry model, has a stenosis length of ten times the arterial radius (Rp = Rd = 3 mm, see Fig. 3) and a diameter reduction of 40% (64% area reduction). In the Reynolds number, a kinematic viscosity of 0.035 cm^2^/s was used, a value nears the asymptote in the Carreau model for blood. Zero velocity was specified on the plaque wall. A stress free boundary condition was specified at the outlet. Adequate distal length was ensured for accurate determination of pressure drops due to the lesion and for the convergence of the calculations. The calculations were done at the mean flow rate Q = 50 mL/min (typical of basal physiological values in a coronary vessel of 3 mm size [26]). Heart rate was 75 beats/min and blood density was 1050 kg/m^3^ [26].

**Figure 4:**
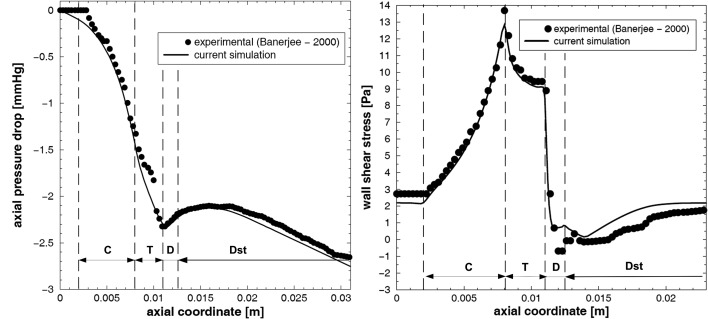
(a) axial pressure drop and (b) wall shear stress distribution along the stenosis during the cardiac cycle for mean flow rate Q = 50 mL/min.

The evolution of the axial pressure drop along the stream-wise direction and the wall shear stress profile is compared to the experimental data presented by Banerjee et al [26] as shown in Figure 4. It can be seen that the present numerical result is in good correlation to the experimental data obtained by Banerjee et al.

## Results and Discussions

In the present study, the characteristics of the flows through the series stenoses were investigated. The geometrical configuration of the stenosis model used in this study has been shown in Figure 1.

### Pressure distribution

**Figure 5:**
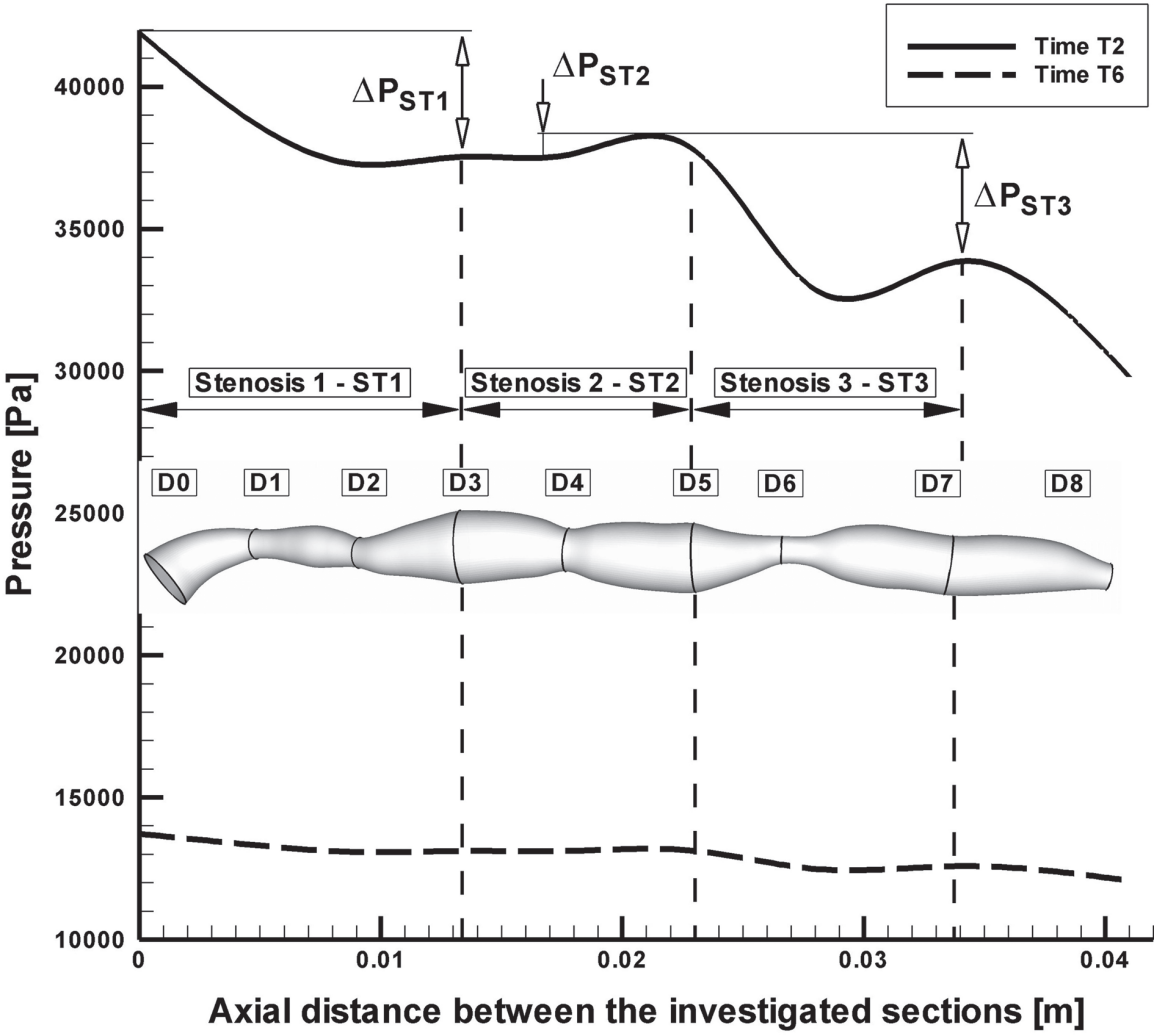
Pressure drop across the investigated multiply stenosed RCA.

The pressure distributions along the axial direction with time for the stenosed RCA are shown in Figure 5. The pressure drop increases with the increase of percentage stenosis. In stenosis ST1 and ST3, the pressure drop is significant during the systolic phase (Time T2) 32.84 mmHg respectively 36.78 mmHg, while during the diastolic phase at the time T6 the pressure drop across the stenoses ST1 and ST3 is lower (4.62 mmHg and 4.81 mmHg). For the stenosis ST2, the pressure drop across the stenosis is lower during the peak systolic phase (1.46 mmHg) and practically is negligible during the diastolic phase (0.03mmHg), (Table 3).

**Table 3:** Hydrodynamic Parameters According to the Stenotic Flow

Stenosis	Time	Q (l/min)	ΔP (mmHg)	Re_throat_	Vmax_throat_	WSSmax	WSSmin
ST1	T2	0.351	32.84	1141	2.27	210	10
	T6	0.113	4.62	369	0.734	45	20
ST2	T2	0.37	1.46	865	1.165	110	10
	T6	0.118	0.03	275	0.371	20	10
ST3	T2	0.35	36.78	1118	2.33	250	10
	T6	0.112	4.81	361	0.752	50	10

Overall pressure drop increased appreciably with flow rate (at the peak systole, Fig. 5). Values of the overall pressure drop increased from 1700 Pa (12.75 mmHg) at the time T6 to 11000 Pa (82.5 mmHg) at the time T2 (Fig. 5).

At the time T2, pressure drop to the minimal throat value (stenosis ST3) increased from 4.81 mmHg at 112 mL/min to 36.78 mmHg at 351 mmL/min (Table 3).

In this study, the wall pressure decreased towards the periphery of the coronary artery tree with elevated pressure drops in stenotic segments. The increased pressure drop in stenoses reflects the elevated energy needed to drive the flow through these regions.

**Figure 6:**
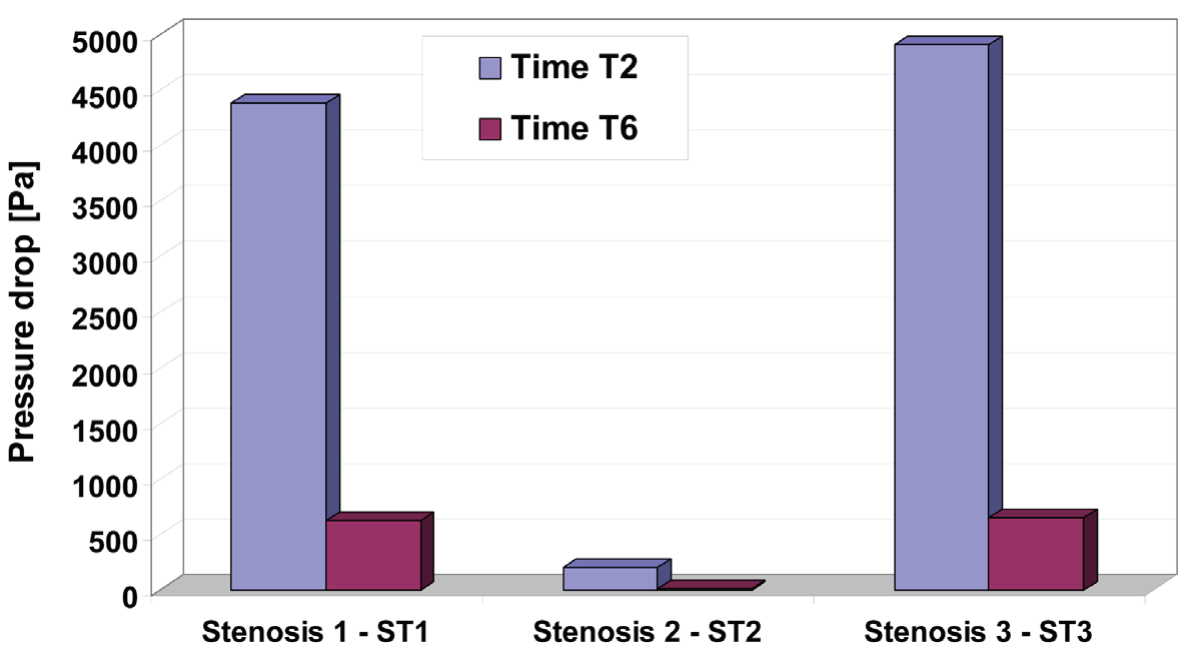
Pressure drop for different time step in the investigated stenosis. Time T2 and T6 correspond to the time steps indicated in Figure 1.

To understand the pressure-flow relationship, pressure drop across the constrictions has been evaluated in Figure 6 for two different time steps. Pressure drop variation depends and varies with increase the number of constrictions and this severity, and also depends on whether the flow is accelerating or decelerating and also on the exact state of these two phases (Table 3, Fig. 6 respectively).

Energy loss associated with such flow expansion after each constriction will be large and consequently the pressure drop will be higher (Fig. 6). The pressure drop associated with spacing between sections D2 and D3, D6 and D7 during the peak flow (time T2) may be attributed to the greater energy loss associated with intense re-circulation zones and associated vortex shedding downstream to the setnosis ST1 and ST3.

### Flow separation and secondary flow

**Figure 7:**
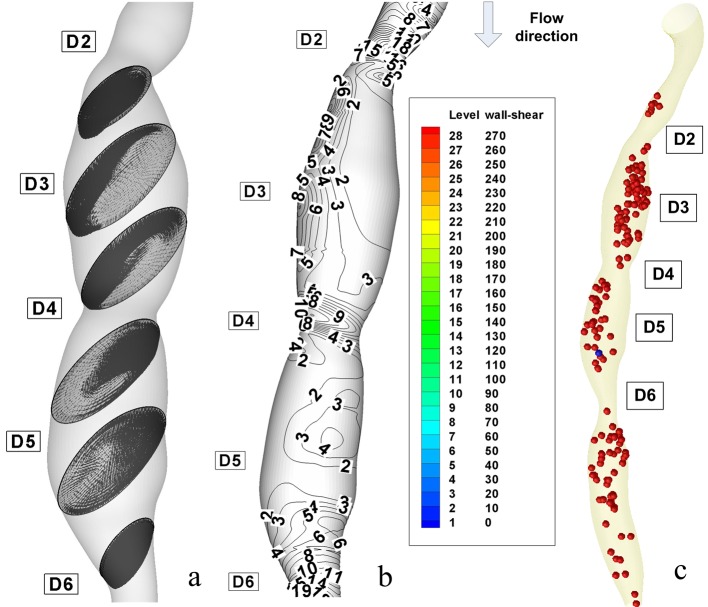
Disturbed flow indicators and computational particle deposition patterns at the peak systole T2 = 0.26 s. (a) cross-sectional transient velocity vector fields; (b) temporal WSS magnitude contours in stenosis ST1 and ST2 (WSS value are in (PA)); (c) particle transport and deposition in the stenosed RCA geometry at the time T2.

**Figure 8:**
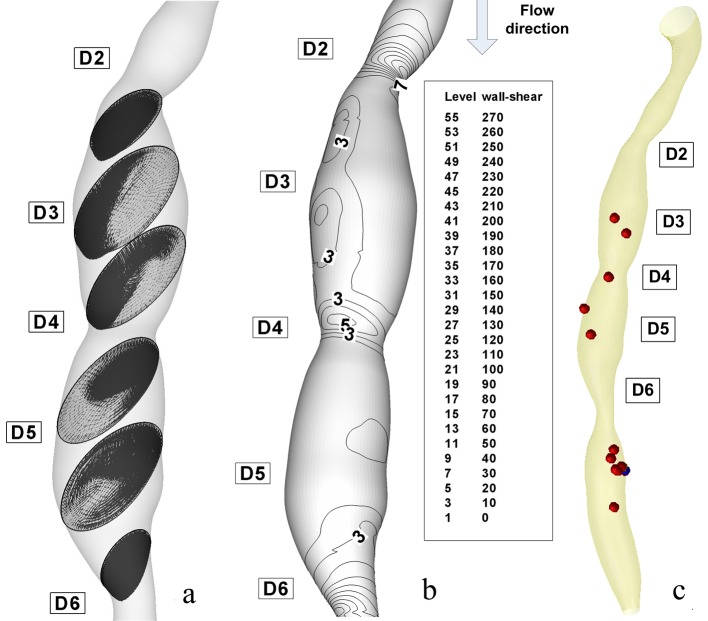
Disturbed flow indicators and computational particle deposition patterns at the end of diastole T6 =1 s. (a) cross-sectional transient velocity vector fields; (b) temporal WSS magnitude contours in stenosis ST1 and ST2 (WSS value are in (Pa)); (c) particle transport and deposition in the stenosed RCA geometry at the time T6.

The flow separation regions can be seen clearly from the instantaneous velocity vector panels which are illustrated in Figure 7a and Figure 8a for different degree of stenosis (54% luminal diameter reduction at the stenosis ST1 and 28% luminal diameter reduction at the stenosis ST2). One can observe the pattern of velocity vector distinctively showing the recirculation zones with the formation of the eddy at the downstream couple of stenoses.

From these figures, it can be observed that the recirculation eddies are formed downstream of the each stenosis. There exists a separation streamline that divides the flow into two regimes one of which is the recirculating region distal to each stenosis, and the other is the main flow field carrying the bulk of the flow near the centre of the tube.

Evidently, the development of the recirculation zone downstream to the each constriction is restricted by the presents of the next stenosis.

The peak velocities from the current simulations are compared with in vivo measured data by Di Mario et al [27] The results from our simulation show that the peak velocity at the throat of the stenosis ST2 is about 1.165 m/s (in 28% diameter reduction stenosis), at the throat of the stenosis ST1 is 2.27 m/s (in 53% diameter reduction stenosis) against a value of 0.78 m/s in healthy arteries (Table 3). We mentioned that our result is in good correlation with both in vivo measured data by Di Mario et al [27] and Siebes et al [28] and the experimentally measured data by Li et al [29].

At the peak flow rate, the Reynolds numbers at the narrowest points in 28%, 53%, and 54% stenoses (stenosis ST2, ST1 and ST3) are about 865, 1141, and 1118, respectively. In the stenosis ST3, the maximum Reynolds number at the throat decreased because of the decrease of flow rate. Downstream of stenosis, flow might become transitional because of the sudden expansion and strong recirculation.

The instantaneous velocity field, WSS and pressure distributions are shown in Figure 7 at time T2 (peak systole) and Figure 8 at the time T6 (end diastole). The recirculation region is non-symmetric and the maximum velocity at throat is around 2.33 m/s for the stenosis ST3. The pressure drop in stenotic arteries is on the throat and it reach 36.78 mmHg, this value is realistic from a physiological point of view [28] (Table 4), and therefore the results are representative for the flow in stenotic arteries.

**Table 4:** Comparison of the Our Results With Experimental and Vivo Data From the Literature

Hemodynamic characteristics	Present study		Experimental Li et al. [29] for 2D model		In vivo data	
	%DS	Value	%DS	Value	%DS	Value
Peak velocity (m/s)	28% - ST2	1.165	30%	1.47	-	-
	53% - ST1	2.27	70%	3.2	^1^69.1 ± 4.8%	2.1 ± 2.8

Reynolds number in the throat (-)	28% - ST2	865	30%	528	-	-
	53% - ST1	1141	70%	1206	-	-

Peak WSS (Pa)	28% - ST2	110	30%	45	-	-
	53% - ST1	210	70%	230	-	-

Pressure drop (mmHg)	28% - ST2	0.03	30%	*8.25	-	-
	53% - ST1	32.84	70%	*39.75	^2^52.2 ± 8.6%	36.9 ± 17.3

%DS percent diameter stenosis; * pressure drop from the inlet to the throat; ^1^Di Mario et al [27]; ^2^Siebes et al [28].

The correct prediction of the vortex dynamics might be important for estimating the near-wall residence times for blood cells. It is particularly relevant because it is now widely accepted that biological processes initiating atherosclerosis are strongly influenced by a combination of fluid and mechanical factors [30].

Figure 7a and Figure 8a shows that the poststenotic deceleration of blood flow induces flow separation and recirculation zones (vortices). Such regions of flow separation and statis are favoured sites for the development of thrombosis and atherosclerosis [31].

### Particle trajectories

**Figure 9:**
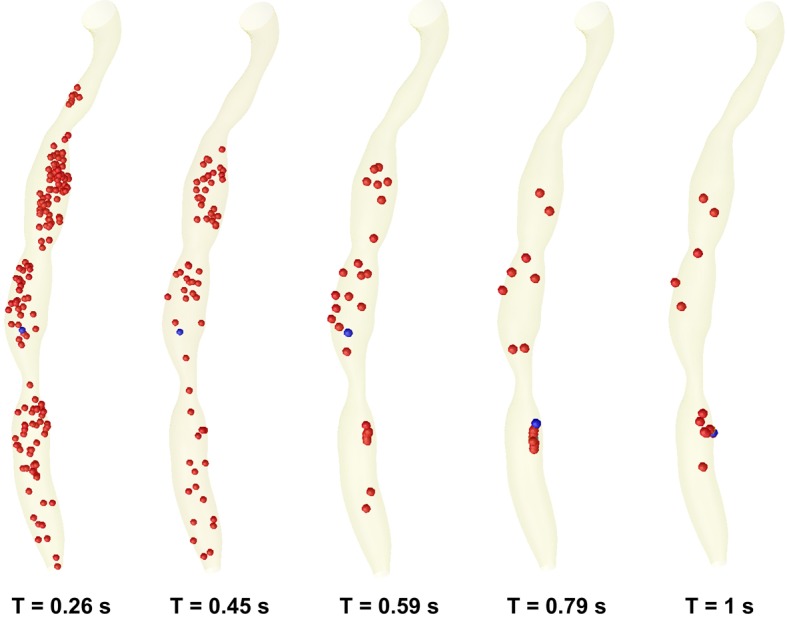
Time evolution of particle transport and deposition in the stenosed RCA at the five critical time levels at the cardiac cycle.

Figure 9 present the time evolution of particle dispersion in the stenosed RCA in form of three dimensional surface projections for a finite volume of particles seeded upstream and then released at t = 0 s in the RCA.

At time t = 0s, it is assumed that a large number of particles (1063 particles) are distributed inside the flow field (the particles is released from the centre of each cells of the inlet section mesh - the number of elements per inlet section is 1063). As time elapses, some particles deposit on the wall, and others are convected downstream (Fig. 9). Particle deposition and accumulation occur along the lateral wall, where flow separation is observed and particle residence time is prolonged (Fig. 9). The high particle deposition region correlates well with low WSS contour areas (Fig. 7c, 8c).

Nevertheless, particle deposition or near-wall aggregation sites are similar to the low WSS regions, which are the regions of the secondary flow toward the wall and the areas of significant changes in velocity-vector direction (Fig. 7c, 8c).

The particle behaviour shows that the distal region near the stenosis wall is a susceptible place for extended particle entrainment, aggregation, and possibly for particle deposition where particle residence time is prolonged (Fig. 9).

Specifically, the expansion of the jet at time level T2 washes a high concentration of particles from the area behind the stenosis. The subsequent growth of the vortex downstream to the stenosis ST1, ST2 and ST3 pushes this elevated particle suspension to the core region of the flow where it is then convected downstream.

**Figure 10:**
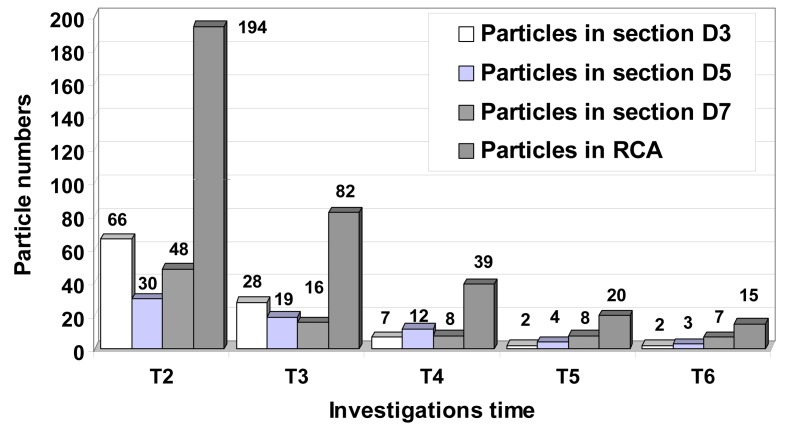
Particle distribution inside to the stenosed RCA during the cardiac cycle. Instantaneous plot of the particle distributions for different time steps in downstream sections of the stenosis ST1, ST2 and ST3. At the end of the cardiac cycle a number of 15 particles (1063 seeded initially at the time T = 0 s) remain inside of the RCA. A number of 7 particles remain in vortical flow patterns result in downstream section of the more sever stenosis ST3.

Due to the severe occlusion and the relatively high Womersley number, platelet-rich zones enter the core of the flow where they are likely to be mixed with thrombin activated by the high shear stresses in the throat. Figures 9 and 10 show a decrease in particles count at the throat and increases in the proximal and distal regions of the serial stenosis. That is in correlation with the conclusions of the other groups [32, 33].

However at the end of one cardiac cycle a percent of 1.4% (15 from 1063 particles) remain inside the stenosed RCA. This percent of particle is enough to initiate the processes of atherosclerosis.

In Figure 7a and Figure 8a, velocity vector plots display a snapshot of the entire flow field at an instant in time and particle trace (the history of a particular fluid element through a region). Of interest are the particles that stay in a recirculation zone or can be come in contact with the wall.

Each of the plots in Figure 9 represents the paths of fluid particles released in the inlet region at the beginning of the cardiac cycle, and the particle depositions at the different time steps of the cardiac cycle.

It would also appear that the particle residence time and particle deposition increase significantly due to the severe occlusion (Fig. 10).

We can see that particle exiting the throat during the decelerating portion of the flow will tend to be washed downstream as the growth of the near-wall vortex pushes them out into the core of the flow. Particle leaving the throat region during the accelerating phase will tend to become entrained in the vortices and remain here for several pulses. Thus, the degree of particle entrainment in conjunction with the strengths and near-wall residence times of the vortices are strong indicators of susceptible sites of secondary stenoses.

### Wall shear stress and intimal thickening

With regard to fluid dynamics, especially in the case of poststenotic regions, the areas of low wall shear stress are also associated with flow separation, that is, a reversal or disturbance of the flow, and a greater fluctuation of wall shear stress (Fig. 7b, 8b). This may be important because it has been suggested that the fluctuation of the wall shear stress or disturbed flow leads to increased endothelial cell turnover and intimal thickening. Importantly, in vivo study has shown that the poststenotic region of subcritical stenoses, for example, less than 60%, is associated with increased intimal thickening, and this intimal thickness also correlated inversely with shear stress [34].

If the blood flow is increased, that increase wall shear stress, an adaptive increase in arterial luminal size is observed. If the blood flow is decreased, usually by a proximal stenosis that decreases the wall shear stress, there is an adaptive decrease in arterial lumen size. This decrease in luminal size elevates the wall shear stress, and the decrease in lumen continues until the wall shear stress is returned to normal [35].

In Figures 7b and 8b, the wall shear stress distribution shows a close reflection of the outline of the stenoses. The point where the maximum shear stress occurs lies at the narrowest cross-section D4 for stenosis ST2 and section D6 for stenosis ST3. In Figure 7a, the separated region at the time T2 occurs at the downstream of the stenosis ST1 and ST2 in sections D3 and D5. There is a rapid increase in wall shear stress upstream of the first stenosis, then after the critical height of stenosis (section D2 and D4), the wall shear stress decreases until separation occurs. In Figure 7a, a larger separation region is observed downstream of the stenosis ST1 and ST3. The maximum value of wall shear stress is at the critical height of the distal stenosis ST3. Figure 7a depicts two large recirculation zones the first is at downstream of the stenosis ST1 until the upstream of the stenosis ST2 and the second is downstream of the distal stenosis ST2. A large separation zone is observed also downstream to the stenosis ST3. The peak of wall shear stress is at the critical height of both stenoses, approximately with the same value.

Figure 7a shows wall shear stress (WSS) distributions at the time T2 = 0.26 s, the time where the flow is at a maximum (peak systole). The range of WSS in this figure varies from 5 to 234 Pa as opposed to 5 to 45 Pa at the time T6 = 1s (Fig. 8b). Three intense regions of low WSS apear downstream at the each stenosis (Fig. 7a, 8a). For the each this regions the WSS is approximately 5 Pa. One the opposide side of this regions we have the regions of moderate and high WSS (WSS varies from 15 to 90 Pa at the time T2 and from 15 to 30 Pa at the time T6).

At the peak velocity (T2) the regions of moderate WSS have intensified to high WSS (up to 90 Pa) and in the regions of the throat the WSS have also intensified reach the value of 210 Pa. At the throat of the stenoses, the maximum WSS is increasing dramatically with the increase of area reduction. The maximum WSS in the stenosis ST1 is about 210 Pa, rise to 110 Pa for the stenosis ST2. One diameter downstream of the each stenosis, the WSS is low because of the formation of the recirculation zone.

As the flow decelerates the regions a high WSS begin to dissipate and more regions of moderate and low WSS begin to appear (time T6, Fig. 8a).

The location of the WSS peaks agrees well with clinical observations of elevated mass transfer in stenoses, i.e., mass transfer occurs on the fore and aft sides of the occlusion and not the throat [36].

From earlier studies by Fry [37], WSS was found to have immediate influence on the endothelial histology and a 40 Pa shear stress is able to damage endothelial cells. Ramstack et al [38] showed that higher WSS (about 100 Pa) would strip the endothelial cells and prevent the endothelium from inhibiting thrombogenesis. Our study shows that the peak WSS reached 110 Pa at the throat of the stenosis ST2 (28% diameter reduction) which is high enough to damage the endothelial cells. In the stenosis ST1 and ST3 (54% and 53% diameter reduction), the peak WSS is 210 Pa and 250 Pa, more than 100 Pa, which suggests that there may be stripping of endothelial cells at the 50% stenosis stage.

As shown in atherosclerotic coronary arteries, regions of flow acceleration were associated with high WSS (Fig. 7, 8).

We observed that the WSS varies from point to point along the irregular geometry of the stenosis. The sudden narrowing of the stenosed lumen leads to characteristic disturbances of the flow profile: high local WSS at the arterial stenosis, poststenotic vortices, and stagnation point. Both wall high shear and stagnant flow have been identified as favourable conditions for platelet aggregation through inherently different pathways [32].

### Study limitations

Some limitations of our study should be pointed out. First, the heart movement and the movement of the coronary arteries due to muscle tension cannot be simulated yet, therefore, not included in the present calculations. Second, we modelled only the trunk of the RCA by ignoring all of its branches. Third, in this model vessel walls are assumed rigid, results might differ in elastic models of the coronary artery wall. Zeng et al [39] have incorporated the effects of physiologically realistic arterial motion into a simulation of blood flow patterns in the RCA. They results show that arterial motion had little effect on the WSS distribution within the RCA, and that flow in the moving artery followed the instantaneous dynamic geometry quite closely. These results agree with the findings of other groups [40].

### Conclusions

The dynamics of the flow describing separation, reattachment, and the formation of recirculation eddy for the above multi-constricted RCA flow are studied through the streamline, velocity, pressure drop, wall shear stress and particle depositions in the present investigations.

Highly accurate anatomy for the generation of geometric models is a principal requirement to perform reliable flow simulations and to make assumptions about mass flow, WSS, and wall pressure.

The results of maximum blood velocities from this study agreed well with published clinical measurement, indicating that the model is physiologically realistic. Results in different degrees of stenoses show that severe stenosis caused considerably large pressure drop across the throat. Maximum wall shear stress reaches a level at which endothelial damage may occur at 30% stenosis by diameter, which is generally not regarded as being clinically significant.

The study also provides awareness that the presence of one stenosis in a coronary artery influences the hemodynamic appearance of the other and, consequently, that treating one lesion will unmask the true severity of the second. As shown in this study, it is possible to calculate this effect quantitatively by measuring pressures at the relevant sites within the artery.

The fluid dynamic interaction of multiple sequential stenoses in coronary arteries is complex, often unexpected, and cannot be adequately assessed by visual interpretation on the coronary angiogram.
